# Joint transformer architecture in brain 3D MRI classification: its application in Alzheimer’s disease classification

**DOI:** 10.1038/s41598-024-59578-3

**Published:** 2024-04-18

**Authors:** Sait Alp, Taymaz Akan, Md. Shenuarin Bhuiyan, Elizabeth A. Disbrow, Steven A. Conrad, John A. Vanchiere, Christopher G. Kevil, Mohammad A. N. Bhuiyan

**Affiliations:** 1 Department of Computer Engineering, Erzurum Technical University, Erzurum, Turkey; 2 Division of Clinical Informatics, Department of Medicine, Louisiana State University Health Sciences Center - Shreveport, Shreveport, LA 71103-4228 USA; 3 Center for Brain Health, Louisiana State University Health Sciences Center - Shreveport, Shreveport, LA 71103-4228 USA; 4 Department of Pathology and Translational Pathobiology, Louisiana State University Health Sciences Center - Shreveport, Shreveport, LA 71103-4228 USA; 5 Department of Pharmacology, Toxicology and Neuroscience, Louisiana State University Health Sciences Center - Shreveport, Shreveport, LA 71103-4228 USA; 6 Department of Neurology, Louisiana State University Health Sciences Center - Shreveport, Shreveport, LA 71103-4228 USA; 7 Department of Psychiatry, Louisiana State University Health Sciences Center - Shreveport, Shreveport, LA 71103-4228 USA; 8 Department of Pediatrics, Louisiana State University Health Sciences Center - Shreveport, Shreveport, LA 71103-4228 USA; 9 Department of Molecular and Cellular Physiology, Louisiana State University Health Sciences Center - Shreveport, Shreveport, LA 71103-4228 USA

**Keywords:** Alzheimer’s disease, MRI, Transfer learning, Sequence classification, Vision transformer, Neurological disorders, Machine learning, Image processing

## Abstract

Alzheimer’s disease (AD), a neurodegenerative disease that mostly affects the elderly, slowly impairs memory, cognition, and daily tasks. AD has long been one of the most debilitating chronic neurological disorders, affecting mostly people over 65. In this study, we investigated the use of Vision Transformer (ViT) for Magnetic Resonance Image processing in the context of AD diagnosis. ViT was utilized to extract features from MRIs, map them to a feature sequence, perform sequence modeling to maintain interdependencies, and classify features using a time series transformer. The proposed model was evaluated using ADNI T1-weighted MRIs for binary and multiclass classification. Two data collections, Complete 1Yr 1.5T and Complete 3Yr 3T, from the ADNI database were used for training and testing. A random split approach was used, allocating 60% for training and 20% for testing and validation, resulting in sample sizes of (211, 70, 70) and (1378, 458, 458), respectively. The performance of our proposed model was compared to various deep learning models, including CNN with BiL-STM and ViT with Bi-LSTM. The suggested technique diagnoses AD with high accuracy (99.048% for binary and 99.014% for multiclass classification), precision, recall, and F-score. Our proposed method offers researchers an approach to more efficient early clinical diagnosis and interventions.

## Introduction

Alzheimer’s disease (AD) is distinguished by the accumulation of aberrant protein deposits in the brain, known as plaques and tangles, which result in the death of nerve cells and the degeneration of brain tissue. Neural degeneration reduces cognitive function and causes mood and behavior changes^1,2^. AD is typically categorized in three stages^3,4^. The first stage is the preclinical stage, characterized by brain, blood, and cerebrospinal fluid (CSF) abnormalities without outward signs^5^. It is believed that AD pathology begins at least 20 years before symptoms appear^6^. The second stage of the disease is referred to as mild cognitive impairment (MCI), which involves cognitive impairment confined to a single cognitive domain, usually memory. Dementia, the final stage of the disease, is defined as a cognitive disturbance in more than one domain, often memory and executive function, with substantial interference with daily life activities.

With the recent approval of new drugs for early AD intervention, early detection of AD and differentiation of MCI have become of primary importance for successful disease treatment and management^7,8^, and to slow disease progression and improve the quality of life for those with AD^9,10^. AD classification is one of the most challenging problems neurologists face^1^. Advances in computer-aided diagnosis (CAD) systems based on neuroimaging data tools have improved classification. CAD systems can be divided into conventional and deep learning-based techniques. Most traditional approaches to image analysis employ a four-stage pipeline of pre-processing, segmentation, feature extraction, and classification^9^. Deep learning (DL) algorithms have an advantage over conventionally based methods because they require little or no image pre-processing. They can automatically infer an optimal data representation from raw images without requiring prior feature selection, resulting in a more objective and less biased process^10–14^. CNN-based architectures are used extensively for medical image analysis. They have been applied to 2D and 3D ultrasound and MRI images^15^ and are the deep models used most frequently to detect AD^15–18^. However, a 3D MRI brain image consists of stacked 2D data slices. 3D-CNN model to learn spatiotemporal features would be optimal, which is impossible with 2D CNN. But, because it requires many parameters and a high amount of computation, the 3D model cannot be used to construct deep models^15^.

Although transformer architecture dominates natural language processing, its use in medical imaging has been limited^19^. However, Vision Transformer (ViT) has recently gained popularity due to its impressive results in various medical imaging tasks, including image classification, object detection, and semantic segmentation^20^. ViT took note of the scaling success of Transformers in NLP and applied a standard Transformer to images with minimal modifications. Transformer-based architectures have also been used in medical image analysis^21,22,24^. ViT has recently demonstrated superior performance in many computer-vision tasks, making it a viable alternative to CNN as a network architecture^23^. CNNs collect features gradually from local to global by adding more convolutional layers. ViT, on the other hand, uses a multi-headed self-attention mechanism to capture long-range dependencies. For this approach, the model equally weights all elements in the input sequence for superior performance. ViT extracts features across the entire image without degrading image resolution, preventing spatial loss from information skipping. Thus, ViT is ideal for brain imaging analysis. The self-attention strategy of ViT has the capacity to accurately capture the interdependencies between various dispersed networks of brain regions^21^.

ViT is based on the concept of Transformers from natural language processing (NLP) applied to medical images. It uses a standard Transformer architecture, with minimal modifications, to process MRI images instead of text. Other neural network models, which process the image sequences sequentially (RNN) or in parallel (CNN), require more time to train and infer the results, and fail to control for long-term dependencies among the image layers^24^. The joint transformer handles long-range dependencies, avoids recursion, and allows parallel computation to reduce training time and avoid performance drops due to long-range dependencies^24^.

ViT has outperformed CNN in several computer-vision tasks, giving it an appropriate network architectural option. We were motivated to use ViT's benefits to diagnose AD patients using 3D MRIs. The lack of large-scale datasets in this field is one of the major obstacles to training deep models from scratch. The model can adapt to the smaller target dataset by using transfer learning to learn from a larger dataset. The 3D data from a plane was divided into a 2D slice array in order to benefit from transfer learning using a pre-trained ViT. Furthermore, it should be possible for slice-based methods to track the dependencies of related features across slices. The sequence classification task uses a time series classification with a transformer to get around this issue. We have combined time series transformers and pre-trained ViT to create a deep learning-based classification system for AD.

The goal of this article is twofold with respect to the proposed ViT: (1) evaluate the predictive performance of ViT combined with a transformer neural network; and (2) capture long-range dependencies and the global context of MRIs, allow parallel computation to reduce training time, and avoid performance drops due to long-range dependencies. We propose testing the hypothesis that ViT with a time series transformer performs better for AD patient classification based on MRI by using the self-attention mechanism to capture long-range dependencies and contextual relationships from MRI images.

## Methods

**Figure 1:**
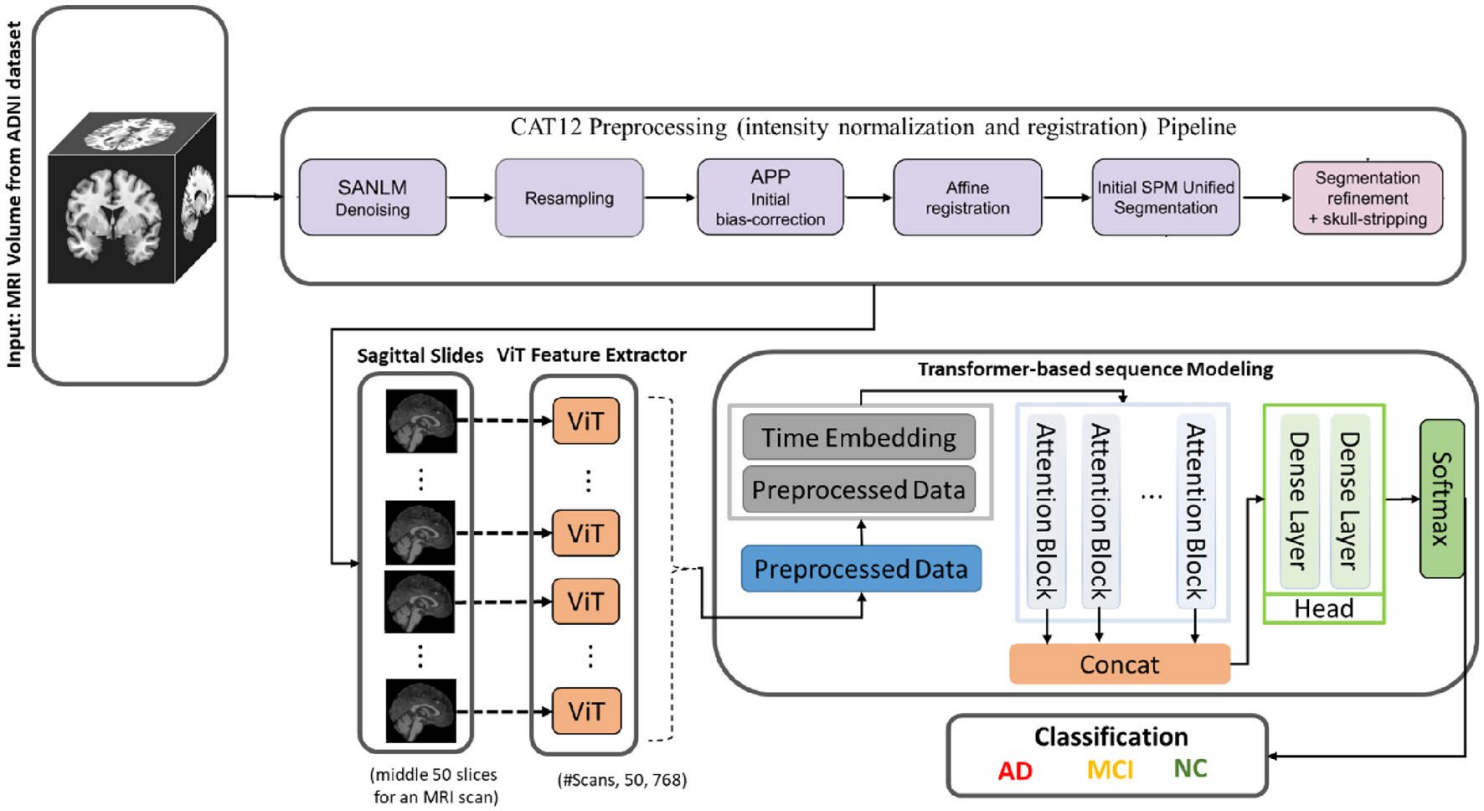
The proposed pipeline of the ViT-TST. MRI images were pre-processed using CAT12 (image registration to standardize the images and skull stripping to reduce biases by ensuring consistent voxel intensities). ViT was used from each plane to derive slice attributes. Finally, the time series transformer was used to classify the feature sequences.

We used ViT to derive T1-weighted MRI slice attributes and a transformer neural network model for sequential feature classification, maintaining inter-association between the slices. The transformer neural network architecture and the ViT architecture for the sequential feature classification model are explained in Supplementary Sections 1 and 1.1. The summary of the ADNI dataset and steps of the proposed method are described in Sections 2.1–2.4, and the pipeline of the proposed method’s architecture is shown in Fig. 1.

### Dataset

Data used in this study was obtained from the Alzheimer’s disease Neuroimaging Initiative (ADNI) database (adni.loni.usc.edu). The ADNI was launched in 2003 as a public–private partnership led by Principal Investigator Michael W. Weiner, MD. The primary goal of ADNI has been to test whether serial magnetic resonance imaging (MRI), positron emission tomography (PET), other biological markers, and clinical and neuropsychological assessment can be combined to measure the progression of mild cognitive impairment (MCI) and early Alzheimer’s disease (AD).

We performed binary and multiclass classification using T1-weighted 3D MRI scans from ADNI^25,26^. We trained and tested the models on subjects who had scans taken at screening and at 6- and 12-month visits (ADNI1: Complete 1Yr 1.5T data) and on subjects who had scans taken at screening, and at 6 months, 1 year, and 18 months (MCI only), and 2 and 3 years (normal and MCI only) (ADNI1: Complete 3Yr 3T data). We tested the model performance in three types of MRI scans (from the top down, axial plane; from front to back, coronal plane; and side to side, sagittal plane). We randomly split each data set into 60% training, 20% testing, and 20% validation sets. We performed extensive experiments on binary (NC/AD) and multi-classification tasks (NC/MCI/AD) to assess the proposed method. The details of model variants are listed in Supplementary Table S1. The configuration of training parameters is summarized in Supplementary Table S2. Both binary and multi-classifications were performed for all sagittal, coronal, and axial planes. We also implemented different baseline architectures to make comparisons with the proposed method. The details of baseline model variants are listed in Supplementary Table S3. The Complete 1Yr 1.5T data results are provided in Supplementary Section 3. The descriptive statistics of the ADNI data are provided in Table 1.

**Table 1:** The details of data collections.

	Image scans#	NC	MCI	AD	Male	Female	Age (years)
ADNI1: Complete 3Yr 3T	351	129	145	77	194	157	75 ± 7.07
ADNI1: Complete 1Yr 1.5T	2294	705	1113	476	1341	953	75 ± 6.6

^#^NC, cognitively normal; MCI, mild cognitive impairment; AD, Alzheimer’s disease.

### MRI Pre-processing

**Figure 2:**
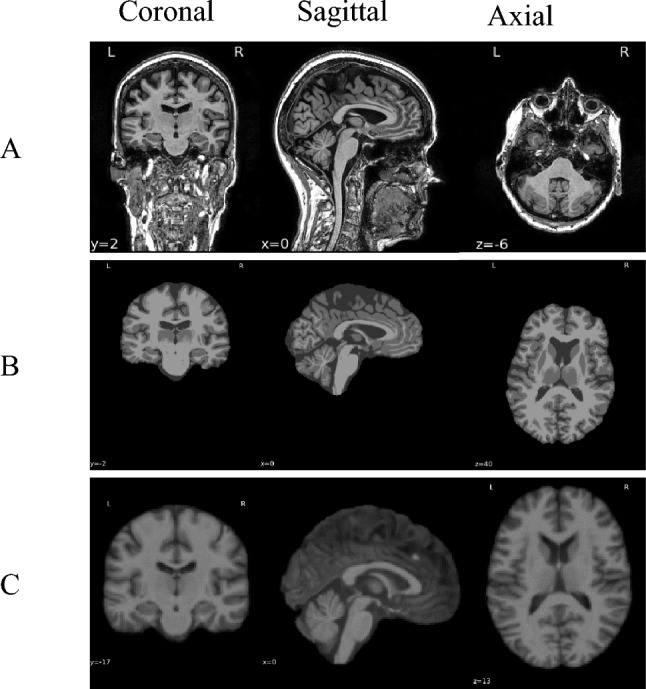
A sample of an MRI slice in three planes (coronal, sagittal, and axial). (**A**) Original MRI; (**B**) segmented anatomical image with the skull removed; and (**C**) segmented anatomical scan warped to MNI space.

T1-weighted MRI scans were standardized in Montreal Neurological Institute (MNI) space. For comparison across subjects each skull was stripped using Statistical Parametric Mapping 12 (SPM12)^27^ and Computational Anatomy Toolbox (CAT12; http://www.neuro.uni-jena.de/cat/) in MATLAB (see Fig. 2).

### Handling 3D MRI using 2D ViT

ViT models are pre-trained using a vast number of 2D data (mageNet21K^28^ and 21,843 classes at a resolution of $224\times 224$ pixels. By taking advantage of transfer learning with a pre-trained 2D network model, we split the standardized 3D MRI into 2D slices. In the 2D slices, the sizes of each slice in the axial, coronal, and sagittal planes were $113\times 137$, $113\times 113$, and $137\times 113$ pixels, respectively.

**Figure 3:**
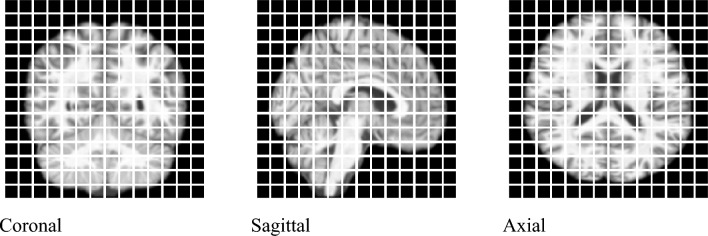
$14\times 14$ image patches. Patches of $16\times 16$ pixels were taken from the input images, with $14\times 14$ patches.

Each slice was then converted to an image of $224\times 224$ pixels, and each slice was divided into $14\times 14$ patches where each patch was $16\times 16$ pixels (see Fig. 3).

**Figure 4:**
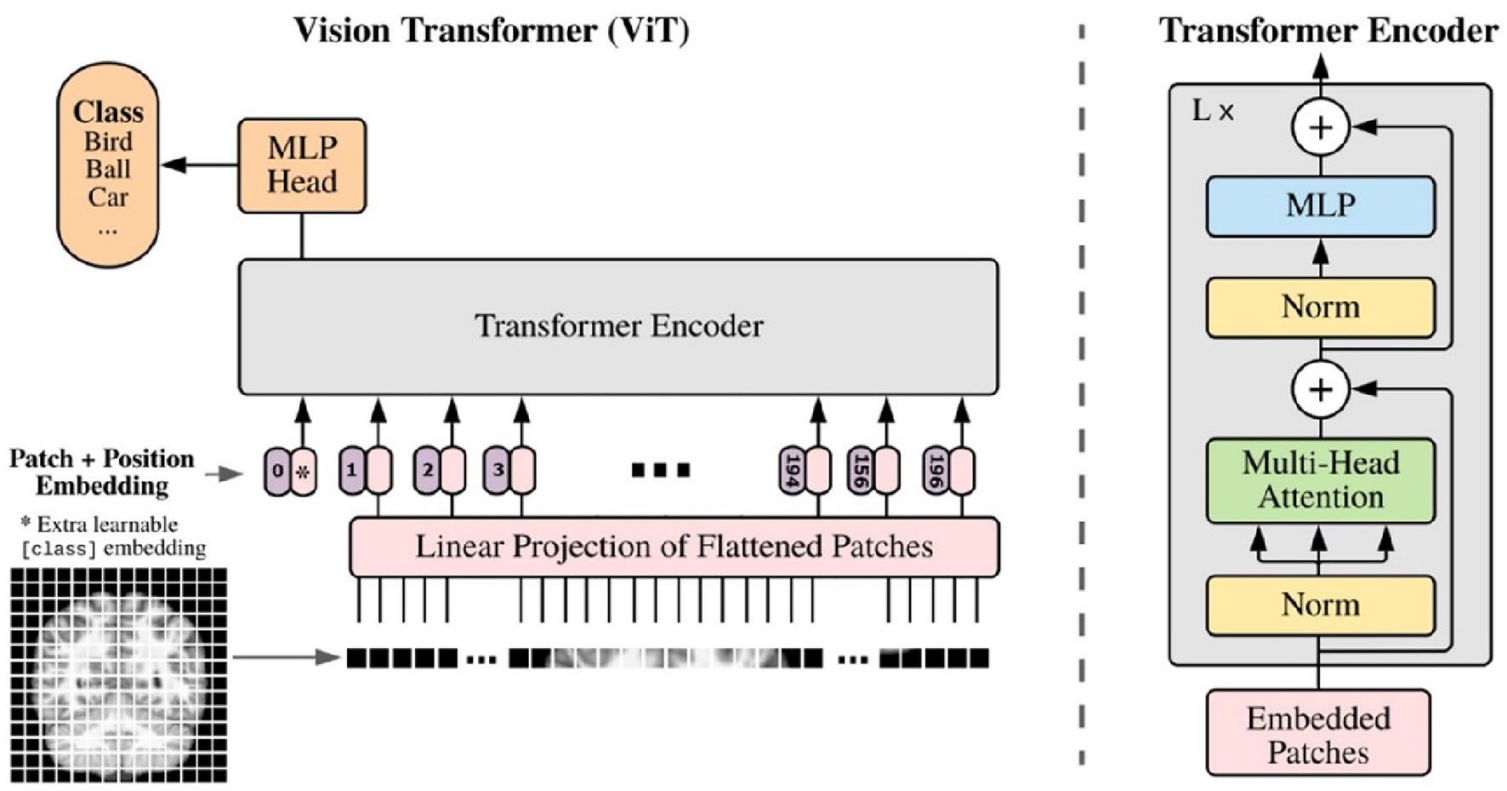
A visual summary of the proposed method. ViTs consist of several transformer blocks. Each transformer block comprises two sub-layers, a feed-forward layer and a multi-head self-attention layer.

These patches, which were flattened into a vector as a sequence of 196 patches, were considered to be an input token for the model, called the self-attention model. Later, we applied a multiple-layer self-attention model and feed-forward neural network to process the sequence of patched pixels and perform the feature selection. The proposed model comprised multiple transformer blocks, each applying the multi-head attention layer as a self-attention mechanism to the patch sequence. Finally, the output of the transformer encoder was processed via a classifier head to produce the final class probability output. Figure 4 provides a visual summary of the proposed model.

### Sequence classification using a time series transformer

**Figure 5:**
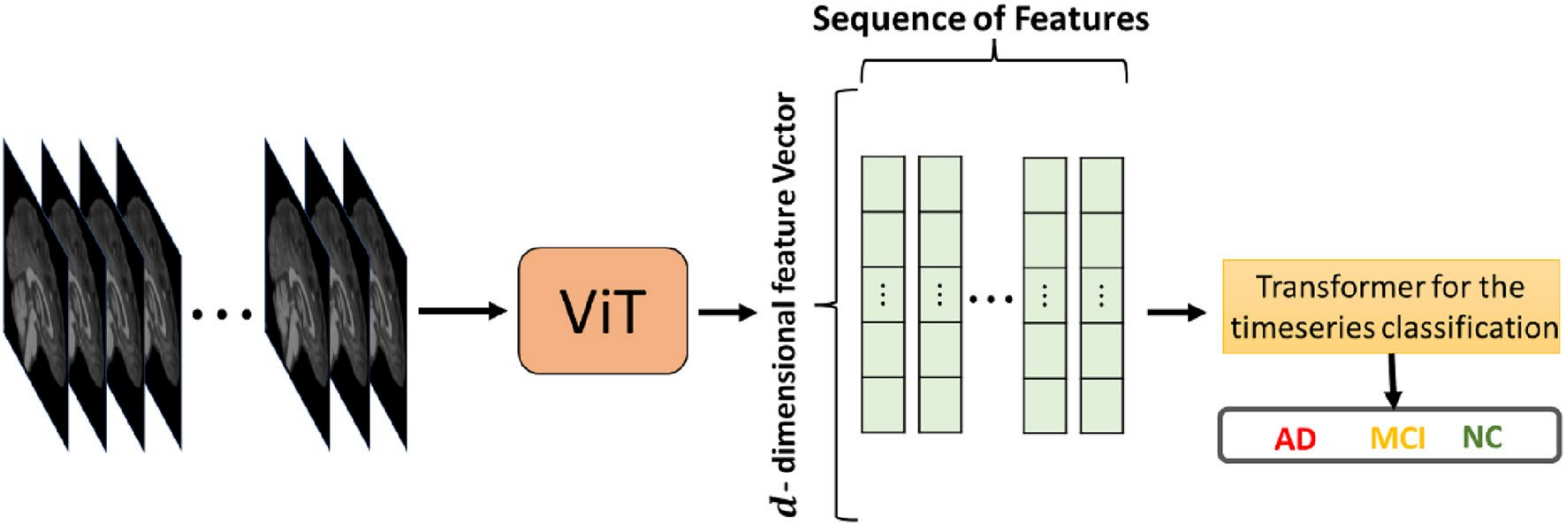
Summary of the proposed model. Features were extracted using ViT and the sequence of features was classified using the time series transformer model.

After features extraction using ViT, a transformer model^24^ was used as a tensor for time-series classification to maintain the relationships between the slices. Due to the self-attention mechanisms of transformer-based time series classifications, long-term dependencies between time steps (features of each slice) were captured more effectively. We then used feature embedding to map each token sequence to a meaningful numeric vector. Since we used ViT to map the slices of each MRI to a sequence of dimensional numerical features, this transformer did not need an embedding module. As each slice provides dimensional numerical features, the MRI classification problem was changed to a multi-dimensional time series classification. The summary of the model is illustrated in Fig. 5.

By utilizing transfer learning with the ViT, we adeptly address the challenges posed by the voluminous nature of 3D MRI datasets. Splitting the 3D MRIs into 2D slices allows for the application of pre-trained models, which are predominantly designed and optimized for 2D image data. This strategy not only circumvents the need for large 3D medical imaging datasets but also uses the power of models trained on extensive 2D image datasets.

Furthermore, our method extends the benefits of transfer learning beyond mere feature extraction from individual slices. By applying transfer learning to the time series classification of these extracted features, we innovatively create a two-tiered application of transfer learning. This approach capitalizes on the inherent temporal information within MRI scans, treating the progression of slices as a sequence that can provide valuable insights into the underlying medical conditions.

This dual application of transfer learning—first, to the feature extraction from 2D slices, and second, to the temporal analysis of these features—ensures a comprehensive utilization of available data. It effectively applies deep learning advances to medical imaging issues, specifically managing 3D MRI data with limited resources and data privacy concerns.

## Results

The analysis was performed in two main steps: feature extraction and sequence modeling tasks on ADNI1: Complete 3Yr 3T and ADNI1: Complete 1Yr 1.5T MRI data. First, we compared our classification result with CNN alongside Bi-LSTM, CNN alongside Transformer, and ViT alongside Bi-LSTM. Then, we compared the classification performance, model accuracy, precision, F-score, and recall. The results for ADNI1: Complete 1Yr 1.5T are shown in the Supplement Section 2.1.

## Experiments on ADNI1: complete 3Yr 3T

### Binary classification

The results from the four architectures were similar, with CNN-TST and ViT-TST achieving the highest accuracy and precision scores of 98.81% and 0.99, respectively. Meanwhile, CNN-Bi-LSTM and ViT-Bi-LSTM had slightly lower scores but still performed well, with accuracy scores of 97.14% and 97.38%, respectively. To measure the ability of our proposed model to capture the positive instances, we calculated the recall score, and to measure the balance between the precision and the recall, we calculated the F-Score. The F-scores and recall scores for all architectures were very similar, ranging from 0.97 to 0.99, indicating good performance in these metrics (see Table 2).

**Table 2:** Results for binary disease classification on ADNI1: Complete 3Yr 3T.

Architecture	ACC	Precision	Recall	F-score
Sagittal				
CNN-Bi-LSTM	97.143% (± 3.658)	0.97	0.97	0.97
CNN-TST	**98.810% (± 2.195)**	0.99	0.99	0.99
ViT-Bi-LSTM	97.381% (± 2.704)	0.97	0.97	0.97
ViT-TST	98.571% (± 2.857)	0.99	0.98	0.98
Coronal				
CNN-Bi-LSTM	**98.333% (± 2.619)**	0.98	0.98	0.98
CNN-TST	97.381% (± 4.185)	0.98	0.97	0.97
ViT-Bi-LSTM	97.619% (± 2.817)	0.97	0.97	0.98
ViT-TST	**98.333% (± 2.827)**	0.98	0.98	0.98
Axial				
CNN-Bi-LSTM	96.190% (± 4.012)	0.96	0.96	0.96
CNN-TST	98.333% (± 2.143)	0.98	0.98	0.98
ViT-Bi-LSTM	98.571% (± 2.182)	0.99	0.98	0.98
ViT-TST	99.048% (± 1.905)	0.99	0.99	0.99

*TST, Time series transformer; ACC, accuracy; Precision, accuracy of positive prediction; Recall, accuracy of positive instances; F1-Score, balance between precision and recall. Significant values are in bold.

The architectures CNN-Bi-LSTM and ViT-TST had the highest accuracy, at 98.333%, while CNN-TST had the lowest accuracy, at 97.381%. Based on precision, F-score, and recall, the CNN-Bi-LSTM and ViT-TST architectures achieved the highest performance, with precision and recall scores of 0.98 and an F-score of 0.98. The CNN-TST also had a strong performance, with a precision and recall of 0.98 and an F-score of 0.97. The ViT-Bi-LSTM model had the lowest precision score, 0.97, but still achieved a strong F-score and recall score of 0.98 and 0.97, respectively.

The table shows the performance of four different deep-learning architectures on a particular task. The ViT-TST architecture performed the best among all the models, achieving the highest accuracy of 99.048% and the highest scores for precision, F-score, and recall. The ViT-Bi-LSTM architecture also performed well, achieving an accuracy of 98.571% and competitive precision, F-score, and recall scores. The CNN-TST architecture attained an accuracy of 98.333% and high precision, F-score, and recall scores. Overall, the ViT-TST and ViT-Bi-LSTM architectures were better suited for the given task, followed by CNN-TST. The CNN-Bi-LSTM architecture was the least effective, although the differences were minor.

The results on sagittal, coronal, and axial planes for binary classification (NC and AD) are listed in Table 2.

**Figure 6:**
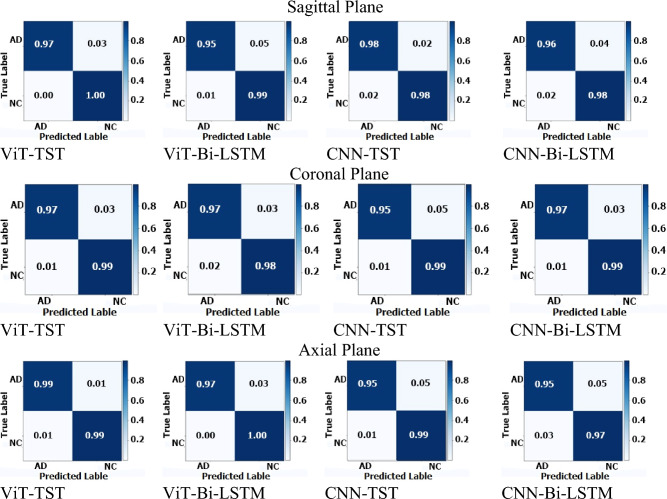
Confusion matrices for binary classification on ADNI1: Complete 3Yr 3T.

To calculate the performance of the proposed model for binary classification, we calculated the confusion matrix based on the proposed method and compared it with the other methods. The performance of the proposed method for binary disease classification using different MRI planes is shown in Fig. 6. The diagonal cells indicate the prediction performance of the models when used to identify the true positive cases. ViT with time series transformer performed better than the other models for all the planes.

### Multiclass classification

ViT-TST architecture achieved the highest accuracy and precision scores for multiclass classification based on sagittal planes. The CNN-Bi-LSTM model achieved an accuracy of 98.028%, with a precision of 0.98, an F-score of 0.98, and a recall of 0.98. The ViT-TST model achieved an accuracy of 98.310%, with a precision of 0.98, an F-score of 0.98, and a recall of 0.98. All four architectures had the best precision score of 0.98, with the ViT-TST architecture achieving the highest accuracy and precision scores (see Table 3).

**Table 3:** Results for multiclass disease patient classification on ADNI1: complete 3Yr 3T.

Architecture	ACC	Precision	Recall	F-score
Sagittal				
CNN-Bi-LSTM	98.028% (± 0.934)	0.98	0.98	0.98
CNN-TST	97.324% (± 2.394)	0.97	0.97	0.97
ViT-Bi-LSTM	97.746% (± 2.456)	0.98	0.97	0.97
ViT-TST	**98.310% (± 1.380)**	0.98	0.98	0.98
Coronal				
CNN-Bi-LSTM	96.479% (± 2.205)	0.96	0.96	0.96
CNN-TST	97.465% (± 2.423)	0.98	0.97	0.97
ViT-Bi-LSTM	97.465% (± 2.164)	0.97	0.97	0.97
ViT-TST	**99.014% (± 1.672)**	0.99	0.99	0.99
Axial				
CNN-Bi-LSTM	96.761% (± 2.601)	0.97	0.96	0.96
CNN-TST	98.169% (± 2.820)	0.98	0.98	0.98
ViT-Bi-LSTM	98.028% (± 1.804)	0.98	0.98	0.98
ViT-TST	**99.014% (± 1.268)**	0.99	0.99	0.99

*TST, time series transformer; ACC, accuracy; Precision, accuracy of positive prediction; Recall, accuracy of positive instances; F1-Score, balance between precision and recall. Significant values are in bold.

For the coronal plane, CNN-Bi-LSTM achieved an accuracy of 96.479%, with a precision of 0.96, an F-score of 0.96, and a recall of 0.96. ViT-TST achieved the highest accuracy, 99.014%, with a precision of 0.99, an F-score of 0.99, and a recall of 0.99. Overall, the CNN-Bi-LSTM architecture had lower scores than the other architectures, especially for accuracy.

For the axial plane, the ViT-Bi-LSTM and CNN-TST architectures also performed well, with accuracy, precision, F-score, and recall values above 0.98. The CNN-Bi-LSTM architecture had the lowest precision and F-score values but achieved high accuracy and recall value. These results suggest that the ViT-TST architecture is the most efficient for this task.

The results on sagittal, coronal, and axial planes for multiclass classification (NC, MCI, and AD) are listed in Table 3.

The ViT-TST algorithm excelled in multiclass classification, demonstrating superiority in true positive (TP), true negative (TN), false positive (FP), and false negative (FN). It consistently demonstrated exceptional performance in accurately classifying instances belonging to various classes, with high TP and TN values across all classes. This demonstrated its ability to accurately identify positive instances and differentiate them from negatives. Additionally, the algorithm minimized false negatives (FN), reducing the risk of misclassifying positive occurrences as negatives, crucial in medical diagnostic applications such as AD classification. The ViT-TST also excelled in managing false positives (FP), ensuring that instances from other classes were correctly identified as non-relevant. Overall, the superiority of ViT-TST in TP, TN, FN, and FP further solidified its position as a highly effective and reliable choice for multiclass disease classification tasks.

**Figure 7:**
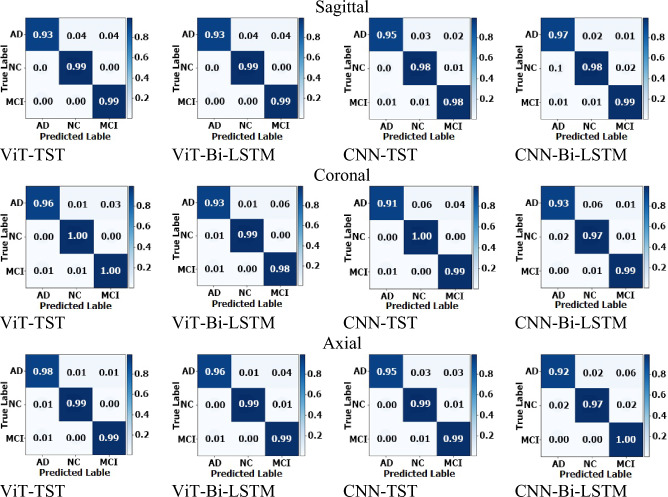
Confusion matrices of axial planes for multiclass disease patient classification (NC, MCI, and AD) on ADNI1: Complete 3Yr 3T.

The performance of the proposed method based on a confusion matrix for multiclass classification on different planes is shown in Fig. 7.

We also compared our proposed model with conventional deep models. Tables 4 and 5 show numerical results from different deep learning model for the binary and multiclassification tasks on ADNI datasets and compared with our proposed model. For binary classification, the proposed method outperformed other traditional models. Moreover, as shown in Table 5, the proposed method ranked first among all competitors.

**Table 4:** Performance compared with state-of-the-art methods for binary disease patient classification.

Work	Input	Image scans	Method	NC/AD classification		
		(NC/MCI/AD)		ACC (%)	SEN (%)	SPE (%)
^29^	Voxel based	429/–/858	3D CNN	90.3	82.4	96.5
^30^	Voxel based	119/233/97	3D DenseNet	88.9	86.6	90.8
^31^	Voxel based	330/299/299	3D CNN	93.2	95.0	89.8
^32^	Patch based	324/316/319	Self-attention	98.0	97.7	98.2
^33^	Voxel based	457/808/346	3D ResNet	94.00	–	–
^34^	Voxel based	407/–/418	3D CNN	99.20	98.90	99.50
^35^	ROI based	209/401/188	2.5D CNN	79.90	84.00	74.80
Proposed method	Sequence based	129/145/77	ViT-TST	99.048	99.5	–
Proposed method	Sequence based	705/1113/476	ViT-TST	95.169	95.5

**Table 5:** Performance compared with state-of-the-art methods for multiclass disease patient classification.

Work	Input	Image scans	Method	NC/MCI/AD classification (%)				
		(NC/MCI/AD)		ACC	Recall	Precision	SPE	F1-Score
^36^	Slice based	300/300/300	VGGNet. 16	91.85	–	–	–	–
^37^	Voxel based	70/70/70	3D CNN	89.10	–	–	–	–
^38^	Slice based	229/243/188	ResNet-18	56.80	–	–	–	–
^39^	Slice based	169/234/101	2D CNN	96.00	96.0	–	98.0	–
^33^	Voxel based	574/808/346	3D ResNet	87.00	–	–	–	–
^40^	Voxel based	207/215/193	3D VGGNet	91.13	–	–	–	–
^41^	Slice based	50/50/50	VGGNet-16	95.73	96	96.33	–	95.66
^42^	Voxel based	351/297/221	3D DenseNets	97.52	97	97.13	–	97.02
^43^	Patch based	475/224/70	3D CNN	97.48	95.33	97.33		97.0
^44^	Slice based	25/13/25	ResNet18 and DenseNet121	98.21	98.14	–	98.14	–
Proposed method	Sequence-based	129/145/77	ViT-TST	99.01	99.0	99.0		99.0
Proposed method	Sequence-based	705/1113/476	ViT-TST	91.42	91.0	92.0	–	90.0

The results on sagittal, coronal, and axial planes for multiclass disease patient classification (NC, MCI, and AD) are shown in Table 5.

The ADNI1: Complete 1Yr 1.5T results are shown in Supplement Section 2.2.1.

## Discussion

In this study, we proposed use of a vision transformer with sequential transformer architecture for binary and multiclass MRI classification. We also compared the performance of the proposed model with other traditional image analysis architectures such as convolutional neural networks and long short-term memory networks on medium and large-sized ADNI datasets.

Reducing false negatives (FN) is crucial in medical diagnosis and healthcare, since models can inaccurately categorize positive occurrences as negative, causing failure to identify or detect cases. In AD categorization, false negative outcomes may misclassify individuals as being in good health, preventing timely identification and administration of appropriate medical interventions. The ViT-TST algorithm appears to be the best choice for binary AD classification. It provides a balanced combination of accuracy and sensitivity, which is crucial for medical applications where both false positives and false negatives can have significant implications. Since minimizing FN is a top priority in AD classification, ViT-TST would be the most suitable choice.

Our proposed method, the ViT-TST, outperformed the ViT-Bi-LSTM, CNN-TST, and CNN-Bi-LSTM models when classifying MCI patients. The ViT-TST achieved higher accuracy, sensitivity, or recall, resulting in an increase in the number of TP predictions and a decrease in the number of FN. This suggests that the ViT-TST accurately identified more MCI patients, reducing the chances that they would be misclassified as healthy or as belonging to other classes. The viability of the ViT-TST for multiclass disease classification tasks, particularly in the diagnosis of AD, is supported by its promising results.With the approval of new drugs for early intervention in AD^7,8^, early detection of AD and differentiation of MCI have become critical. Classifying MCI vs. NC or AD in a multiclassification framework is challenging because it is a heterogeneous condition with many subtypes and causes. Many studies have overlooked this problem and have experimented only with binary classification. However, our experiments were performed on binary (NC/AD) and multiclass disease classification tasks (NC/MCI/AD), and the performance of each model was evaluated using accuracy, precision, F-score, and recall.

When averaging the accuracy rates over all planes for binary disease classification on the ADNI1: Complete 3Yr 3T data, ViT-TST achieved the highest average accuracy of 98.65% (see Table 2), followed closely by CNN-TST with 98.17%. ViT-Bi-LSTM and CNN-Bi-LSTM also performed well, with average accuracies of 97.85% and 97.22%, respectively (see Table 2). These results suggest that all four architectures are effective for binary disease patient classification on this dataset, with CNN-Bi-LSTM being the most effective.

When averaging the accuracy rates over all planes for multiclass disease classification on the ADNI1: Complete 3Yr 3T, ViT-TST achieved the highest average accuracy of 98.77%, followed by ViT-Bi-LSTM with 97.74%. CNN-TST and CNN-Bi-LSTM also performed well, with average accuracies of 97.64% and 97.08%, respectively (see Table 3). These results suggest that all four architectures are effective for multiclass classification on this dataset, with ViT-TST being the most effective.

Evaluation of all the results obtained revealed the ViT-TST architecture to be consistently among the top-performing architectures across all datasets and classification tasks. Therefore, the ViT-TST architecture may be a good choice when designing a classification model for similar datasets and tasks if computational resources and other practical considerations are allowed.

Thus, we have devised a way to classify AD based on deep learning by combining pre-trained ViT and time series transformers. Many tasks have limited data, making training a model from scratch difficult. In small or unbalanced target datasets, transfer learning lets the model learn from a large dataset and adapt to the smaller target dataset. Transfer learning in ViT improves generalization, training speed, and adaptability to datasets and tasks; the proposed method generalizes well when trained on insufficient amounts of data. To take advantage of transfer learning with a pre-trained ViT, the 3D data from a plane were split into a 2D slice array. The problem with slice-based approaches such as CNN is that they fail to retain the dependencies of associated features between slices. To overcome this problem, the sequence classification task uses a time series classification with a transformer. Another alternative is to use 3D deep models; however, transfer learning with a pre-trained 3D is not currently available. Therefore, these approaches do not generalize well when trained on insufficient data.

## Conclusions

Overall, our results show that all four architectures achieved high levels of accuracy and performance on binary and multiclass disease classification tasks. Based on accuracy scores, all models performed well. However, we conclude that the Vi-TST and ViT-Bi-LSTM models perform better than the CNN-TST and CNN-Bi-LSTM models in terms of long-term dependencies among the spatial and temporal patterns in dynamic MRI sequences. In addition, they capture the global context using the self-attention mechanism to ensure that the relevant information from the entire or sequence of images is considered during classification, which reduces overfitting. Transfer learning with the ViT can efficiently handle large 3D MRI datasets by splitting them into 2D slices and applying pre-trained models. This approach not only minimizes the need for large datasets but also utilizes models trained on extensive 2D image datasets. The method also extends transfer learning beyond feature extraction to time series classification, providing valuable insights into underlying medical conditions. We gain deeper understanding of the underlying medical conditions by leveraging the attention mechanism in both feature extraction and classification, demonstrating a sophisticated combination of advanced AI techniques for medical image analysis.

### Supplementary Information

Supplementary Information.

## Data Availability

No datasets were generated or analysed during the current study.
