# Supplementary material for: Joint transformer architecture in brain 3D MRI classification: its application in Alzheimer’s disease classification
Source: Sci Rep. 2024 Apr 18;14:8996. doi: 10.1038/s41598-024-59578-3 (PMC11026447; doi:10.1038/s41598-024-59578-3)
Supplement: Supplementary file 1 — Supplementary Information. [file 41598_2024_59578_MOESM1_ESM.docx]

**Joint transformer architecture in brain 3D MRI classification: its application in Alzheimer’s disease classification**

Sait Alp^a^, Taymaz Akan^b,c^, Md. Shenuarin Bhuiyan^d^, Elizabeth A. Disbrow^c,e,f,g^, Steven A. Conrad^b^, John A. Vanchiere^b,h^, Christopher G. Kevil^d,i^, and Mohammad A. N. Bhuiyan^b,c *^

^a^ Department of Computer Engineering, Erzurum Technical University, Erzurum, Turkey

^b^Department of Medicine, Louisiana State University Health Sciences Center at Shreveport, Shreveport, LA 71103, USA

^3^Center for Brain Health, Louisiana State University Health Sciences Center at Shreveport, Shreveport, LA 71103, USA

^d^Department of Pathology and Translational Pathobiology, Louisiana State University Health Sciences Center at Shreveport, Shreveport, LA 71103, USA

^e^Department of Pharmacology, Toxicology & Neuroscience, Louisiana State University Health Sciences Center at Shreveport, Shreveport, LA 71103, USA

^f^Department of Neurology, Louisiana State University Health Sciences Center at Shreveport, Shreveport, LA 71103, USA

^g^Department of Psychiatry, Louisiana State University Health Sciences Center at Shreveport, Shreveport, LA 71103, USA

^h^Department of Pediatrics, Louisiana State University Health Sciences Center at Shreveport, Shreveport, LA 71103, USA

^i^Department of Molecular and Cellular Physiology, Louisiana State University Health Sciences Center at Shreveport, Shreveport, LA 71103, USA

***Correspondence to:** Mohammad Alfrad Nobel Bhuiyan, PhD, Division of Clinical Informatics, Department of Medicine, Louisiana State University Health Sciences Center at Shreveport, PO Box 33932, Shreveport, LA 71130-3932. Email: Nobel.Bhuiyan@lsuhs.edu

# Transformers

The term ‘transformer’ first appeared in the field of machine translation. The Transformer Neural Network is an attempt to create an architecture that can efficiently deal with long-range dependencies, avoid recursion, and allow parallel computation to reduce training time and avoid performance drops due to long-range dependencies [1]. In natural language processing (NLP), a Transformer comprises two parts: Encoder and Decoder. The encoder turns the input (a sequence of words) into hidden layers, and the decoder turns the hidden layers back into sequences of words in natural language [2]. Self-attention-based architectures, in particular transformers, were built entirely on attention mechanisms. Neither reiteration nor rephrasing plays any role. NLP, computer vision, and audio processing are some of the areas in which transformers have revolutionized deep learning. The critical features of transformers consist of Non-sequential, Self-Attention, and Positional embeddings [1].

Transformers were invented for machine translation to translate sentences from one language to another (See Figure S1 (a)). The core of a transformer consists of a series of encoder and decoder layers. Encoder and decoder layers have their respective embedding layers for their corresponding inputs (Figure S1 (b and c)). The Transformer needs to know the meaning of each word and where it falls in the sequence. Word embedding represents words as real-valued vectors in a vector space such that similar words have similar encoding. The position encoding layer represents the position of a word in the sequence. The outputs of input Embedding are fed into the encoder. In the encoder, they first pass through a self-attention layer where they pay attention to themselves. In other words, the self-attention layer computes the relationship between tokens in an input sequence. Next, a position-wise feed-forward neural network (FFN) receives the output of the self-attention layer. Subsequently, a decoder with the same two layers as the encoder and an attention layer helps it focus on the most crucial parts of the input sequence. The output of the FFN is then sent upward to the subsequent Encoder (See Figure S1 (d)). The output of the final encoder is sent to each decoder in the decoder stack. It is worth mentioning that the structure of the Decoder is very similar to that of the Encoder, except that an additional multi-head attention layer, the Encoder-Decoder attention layer, is included in the decoder. The final Decoder in the stack feeds its output to the Output module, which transforms it into the output sentence.

| 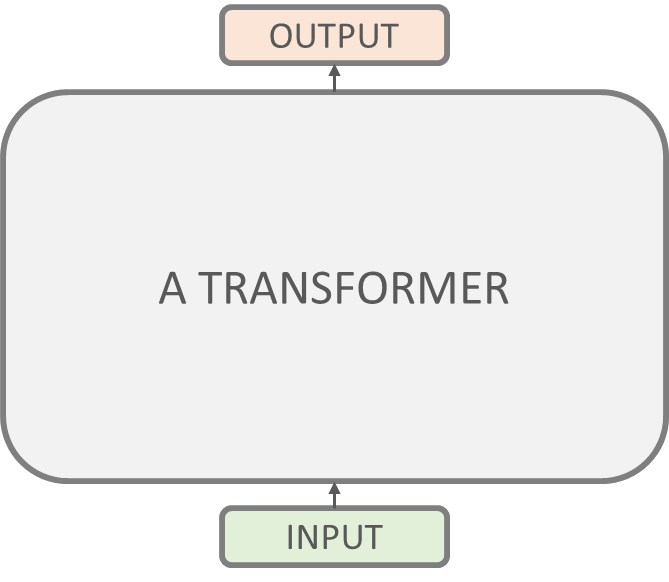 | 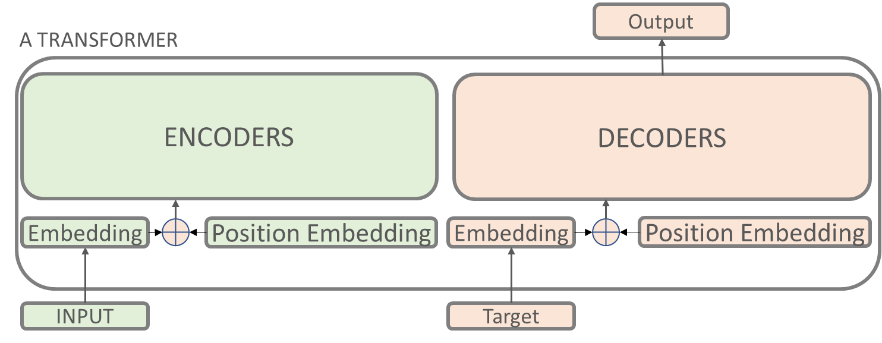 |
| --- | --- |
| (a) | (b) |
| 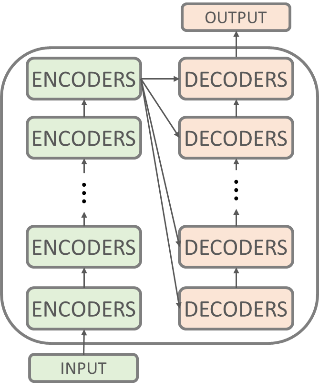 | 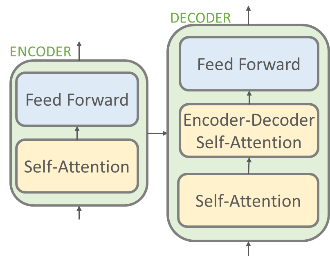 |
| (c) | (d) |
| **Figure S1.** General overview of transformers. Each encoder has two main sublayers, a multi-head attention layer and a fully connected FFN. | |

This module consists of two consecutive layers, the linear and SoftMax layers. The Word Scores, which represent the likelihood of each word appearing in the target vocabulary, are projected from the Decoder vector by the Linear layer. The output sequence of the Transformer is created by the SoftMax layer, which converts these scores into probabilities and maps them to the appropriate word in the vocabulary.

The self-attention mechanism is the foundation of a Transformer’s groundbreaking performance, which associates every word in the input sequence with every other word. Without self-attention, the relationships between words cannot be discovered in the NLP. In addition, in a time-series analysis, finding relationships across time is not possible without self-attention. A single-head attention layer can create a single context for all tokens. However, generating different contexts from one sequence is desired. Multi-head attention is a module for attention mechanisms that runs a different attention mechanism through a different head. This allows self-attention to work in parallel, which means that the same tokens can pay attention to one other from different points of view.

## ViT

The motivation behind ViT is the notable success of transformers in NLP. Therefore, in ViT, slight changes have been made to the standard transformer to enable the same performance with images. As input, the standard Transformer accepts a 1D sequence of token embeddings. The image is divided into patches handled in the same manner as visual tokens. To manage 2D images, $\mathbf{x}\in\mathbb{R}^{H\times W\times C}$ the image is flattened into a sequence of 2D patches $\mathbf{x}_{p}\in\mathbb{R}^{N\times\left( P^{2}\cdot C \right)}$, where (H; W) represents the size of the image, C represents the number of color channels, (P; P) represents the size of each image patch, and $N=HW/P^{2}$ is the number of patches. Next, each patch is turned into a vector and then projected linearly into tokens:

| $\hat{\mathbf{x}}=\left[ {x_{class},x}_{1}\mathbf{E},x_{2}\mathbf{E},\ldots,x_{N}\mathbf{E} \right],\mathbf{E}\in\mathbb{R}^{CP^{2}\times D},$ | (1) |
| --- | --- |

where D is the dimension of the embedding. After that, a positional embedding known as $\mathbf{E}_{\text{pos }}$ is added so that the patches can maintain the information regarding their positions:

| $\mathbf{z}_{0}=\hat{\mathbf{x}}+\mathbf{E}_{pos},\mathbf{E}_{pos}\in\mathbb{R}^{(N+1)\times D}$ | (2) |
| --- | --- |

The tokens are then fed into a Transformer encoder, which consists of L L-base blocks stacked on top of one another. Each base block has a multi-head self-attention block and a multi-layer perceptron (MLP) block with Layer Norm (LN), which is formulated as follows:

| $\mathbf{z}_{\mathcal{l}}^{'}=\mathrm{MSA} \left( \mathrm{LN}\left( \mathbf{z}_{\mathcal{l-}1} \right) \right)+\mathbf{z}_{\mathcal{l-}1}\mathcal{, l=}1\ldots L$ | (3) |
| --- | --- |

| $\mathbf{z}_{\mathcal{l}}=\mathrm{MLP} \left( \mathrm{LN} \left( \mathbf{z}_{\mathcal{l}}^{'} \right) \right)+\mathbf{z}_{\mathcal{l}}^{'}\mathcal{, l=}1\ldots L$ | (4) |
| --- | --- |

| $\mathbf{y}=\mathrm{LN}\left( \mathbf{z}_{L}^{0} \right)$ | (5) |
| --- | --- |

Unlike the original transformer, the ViT does not have a decoder module.

## Transformer model for time-series classification

Transformer models have proven effective in numerous NLP applications, including translation and text classification. Due to its strong capability for capturing long-term dependencies, a Transformer is effective in various time-series classification tasks [3]. By treating the time series as a sequence of tokens, researchers have recently applied Transformers to the classification of time series.

The vanilla transformer was first developed for machine translation. The aim was to translate a sentence from one language to another. It mainly consisted of a stack of encoder and decoder layers. The Encoder mapped the input (language sequence) into hidden layers, and the Decoder mapped the output (natural language sequences). The classification application has no sequence output, unlike the machine translation application. Because of this, the decoder is not used in the transformers.

Viewed from a mathematical perspective, a time series comprises $T$ time steps and $R$ classes. Each time step $t$ in the time series has $d$ features. The time series can be represented as a matrix $X$ with dimensions $T \times d$, where each row represents a time step, and each column represents a feature. It takes as input the time series matrix $X$ and outputs an attention matrix with the exact dimensions. There are several encoders in the encoder stack. Each encoder consists of a multi-head attention layer and a feed-forward layer. A series of feed-forward layers and self-attention layers make up the encoder of the transformer model. The self-attention layer models the temporal dependencies between various time steps in the time series. Embedding and Position Encoding feed the first encoder. The previous Encoder feeds the stack.

Each encoder has two major sublayers, a multi-head attention layer and a position-wise fully connected FFN (Figure S1 (d)). Along with these two sublayers, it also has Residual skip connections around both layers and two Layer Norm layers (see Figure S2).

| 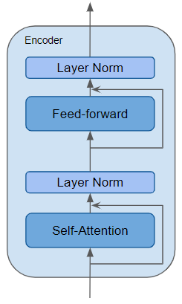 |
| --- |
| **Figure S2**. The layers of self-attention in the Encoder, showing residual skip connections around both layers and two Layer Norm layers. |

In the self-attention layer, attention weights are computed between each time step and every other time step in the time series. These attention weights are between 0 and 1. A scaled dot-product attention function converts a query vector ($Q$) and a set of key-value ($K$) pairs into an output vector ($V$). The importance of each time step in representing the temporal patterns of the time series is reflected in these attention weights. The following equation is used to calculate the attention weights (output matrix):

| $head=\mathrm{attention} \left( Q,K,V \right)=\mathrm{softmax} \left( \frac{\mathrm{QK}^{\top}}{\sqrt{d_{k}}} \right)V$ | (6) |
| --- | --- |

The attention weights are normalized by the SoftMax function so that their sum is 1. The square root of $d_{k}$ is a scaling factor that aids in stabilizing the gradients during training. Unlike the sequence-to-sequence transformer, which had the same Q, K, but a different V, in the time-series classification transformer, all the matrices are identical.

A multi-head attention layer comprises parallel scaled dot-product attention layers, each referred to as a head. The outputs from the H heads are then added and projected onto another dense layer to create the final hidden representation. The multi-head attention function is as follows:

| $\mathrm{multihead} \left( Q,K,V \right)=\mathrm{concat} \left( \mathrm{head}_{1},\mathrm{head}_{2}\cdots\mathrm{head}_{h} \right)W^{O}$ | (7) |
| --- | --- |
| $\text{ head }_{i}=\text{ attention }\left( \mathrm{QW}_{i}^{Q},\mathrm{KW}_{i}^{K},\mathrm{VW}_{i}^{V} \right)$ | (8) |

where $\mathrm{QW}_{i}^{Q},\mathrm{KW}_{i}^{K},$ and $\mathrm{VW}_{i}^{V}$ are weight matrices that map the input matrix $X$ to the query, key, and value spaces. Lastly, the feed-forward layer output is passed through a SoftMax function to generate a probability distribution over the R classes. Based on the class with the highest probability, the time series class label is predicted.

# Experimental setting

All the experiments were carried out using Python on an Intel (R) Core (TM) i5-6400 CPU @ 2.70GHz 2.71, 16 GB RAM, and 8GB NVIDIA GeForce RTX 2080 running Windows 10. The models were implemented using Transformers, Scikit-Learn libraries, and Keras on top of TensorFlow.

The model was trained by Adam optimizer for 100 epochs with a batch size of 32 and a learning rate of 1e-4. Sparse Categorical Cross-entropy was used as a loss function. Also, in the final layers, SoftMax classifiers were used (Table S1).

| **Table S1.** Details of ViT and Time Series Transformer model variants | |
| --- | --- |
| Parameter name | Values |
| **ViT** |  |
| Model | ViT-base-patch16-224-in21k |
| Trained with | ImageNet-21k (14 million images; 21,843 classes) at resolution 224 x 224. |
| Path resolution | $16\times16$ |
| Layers | 12 |
| Hidden size *D* | 768 |
| MLP size | 3072 |
| Heads | 12 |
| Params | 86M |
| Size of Pooler output (feature extractor) | $1\times768$ |
| **Time series Transformer** |  |
| Heads | 10 |
| Encoder layers | 8 |
| Layers | 79 |
| MLP Unit | [100, 30] |
| Input dimension | $50\times768$ |
| Output dimension | #classes (2/3) |
| Params | 14,212,639 |

In addition, as the call-back, the model checkpoint was set to save only the best solution found during training based on the validation’s loss function evaluation. This was accomplished by monitoring the metric on a validation set during training and saving the model checkpoint only when the metric improved. The model checkpoint was saved when the metric improved on a validation set during training (Table S2).

| **Table S2.** Training hyper-parameters for controlling the learning process of classification | | |
| --- | --- | --- |
| Parameter names |  | Values |
| Epoch |  | 100 |
| Batch size |  | 32 |
| Adam Optimizer | learning rate | 1e-4 |
|  | $\beta_{1}$ | 0.9 |
|  | $\beta_{2}$ | 0.999 |
|  | $\epsilon$ | 1e-07 |
| Regularization | Dropout probability | 0.15 |
| Loss function |  | Sparse Categorical Cross entropy |
| classifiers |  | SoftMax |

The details of baseline model variants are listed in Table 3.

| **Table S3.** Details of baseline models (CNN and LSTM) variants | | |
| --- | --- | --- |
| Parameter name | | Values |
| **CNN** | |  |
| Model | | ResNet-101 |
| Trained with | | ImageNet-1k (contains 1,200,000 images) at resolution 224 x 224. |
| Params | | 44.7M |
| Depth | | 209 |
| Size of the last layer (feature extractor) | | $1\times2048$ |
| **Bi-LSTM** | |  |
| Layers | | 6 |
| Hidden Units | | 64 |
| Epoch | | 100 |
| Batch Size | | 25 |
| Adam Optimizer | learning rate | 1e-4 |
|  | $\beta_{1}$ | 0.9 |
|  | $\beta_{2}$ | 0.999 |
|  | $\epsilon$ | 1e-07 |
| Loss Function | | Sparse Categorical Cross entropy |
| Dropout | | 0.15 |
| Activation | | tanh |
| Input Dimension | | $50\times2048$ |
| Output Dimension | | #classes (2/3) |
| Params | | 538,515 |

## Experiments on ADNI1: Complete 1Yr 1.5T

### Binary classification

The results on sagittal planes for binary classification show that among the four models, CNN-Bi-LSTM performed the best with an accuracy of 96.314% (±1.039), followed closely by ViT-TST with an accuracy of 95.000% (±2.151). Both models achieved high precision, F-score, and recall, indicating they are well-suited for binary classification on sagittal planes. The other two models, CNN-TST and ViT-Bi-LSTM, also performed reasonably well, with accuracy scores of 94.492% (±2.169) and 94.364% (±2.085), respectively. However, their precision, F-score, and recall scores were slightly lower than those of the top-performing models.

On coronal planes, ViT-Bi-LSTM achieved the highest average accuracy (94.958%), followed closely by CNN-TST (94.195%), CNN-Bi-LSTM (94.237%), and ViT-TST (94.153%). The precision, F-score, and recall of all algorithms were also quite high, achieving over 94% for each metric. However, CNN-TST achieved a similar accuracy (94.195%) with a lower standard deviation, indicating that it may be a more stable algorithm overall.

The axial plane results clearly show that all four algorithms perform well, with accuracy ranging from 94.66% to 95.68%. The ViT-Bi-LSTM algorithm has the highest accuracy (95.68%) and F-score (0.96). The highest precision and recall values are 0.96. The ViT-TST algorithm has the second-highest accuracy of 95.17%, with precision, F-score, and recall of 0.95. CNN-TST outperforms CNN-Bi-LSTM with 94.78% accuracy. Both algorithms have 0.95 precision, F-score, and recall. The best axial plane algorithm is ViT-Bi-LSTM, followed by ViT-TST (see Table S3). The results for binary classification (NC and AD) on the ADNI data set are shown in Table S3.

**Table S4.** Results for binary classification on ADNI1: Complete 1Yr 1.5T

| Plane | Architecture | ACC | Precision | F-score | Recall |
| --- | --- | --- | --- | --- | --- |
| Sagittal | CNN-Bi-LSTM | **96.314% (±1.039)** | 0.96 | 0.96 | 0.96 |
|  | CNN-TST | 94.492% (±2.169) | 0.95 | 0.94 | 0.94 |
|  | ViT-Bi-LSTM | 94.364% (±2.085) | 0.94 | 0.94 | 0.94 |
|  | ViT-TST | 95% (±2.151) | 0.95 | 0.95 | 0.95 |
| Coronal | CNN-Bi-LSTM | 94.237% (±1.058) | 0.94 | 0.94 | 0.94 |
|  | CNN-TST | 94.195% (±1.818) | 0.94 | 0.94 | 0.94 |
|  | ViT-Bi-LSTM | **94.958% (±1.346)** | 0.95 | 0.95 | 0.95 |
|  | ViT-TST | 94.153% (±2.725) | 0.94 | 0.94 | 0.94 |
| Axial | CNN-Bi-LSTM | 94.661% (±1.124) | 0.95 | 0.94 | 0.94 |
|  | CNN-TST | 94.788% (±1.597) | 0.95 | 0.95 | 0.95 |
|  | ViT-Bi-LSTM | **95.678% (±1.931)** | 0.96 | 0.96 | 0.96 |
|  | ViT-TST | 95.169% (±1.006) | 0.95 | 0.95 | 0.95 |

We also calculated each model’s classification error rate (confusion matrix). Figure S3 depicts the confusion matrices of all the planes for binary classification.

| Sagittal Plane | | | |
| --- | --- | --- | --- |
| 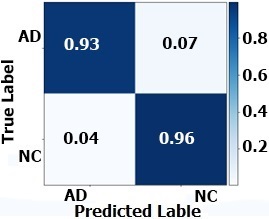 | 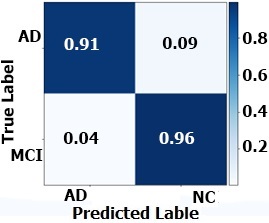 | 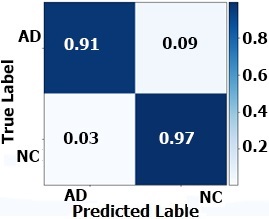 | 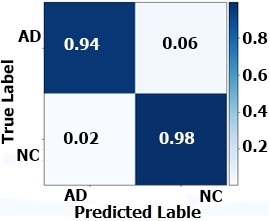 |
| Coronal Plane | | | |
| 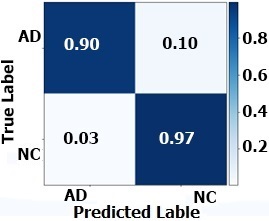 | 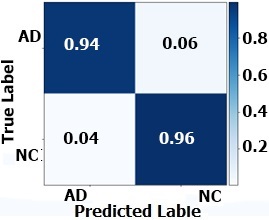 | 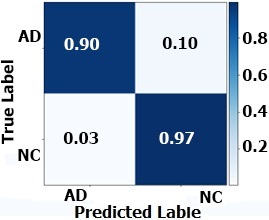 | 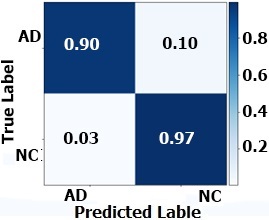 |
| Axial Plane | | | |
| 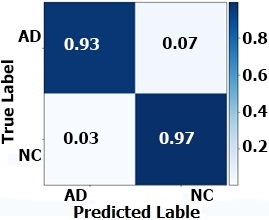 | 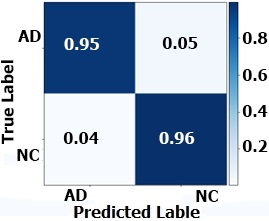 | 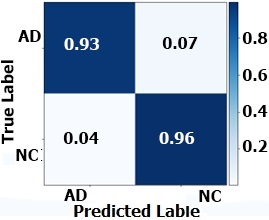 | 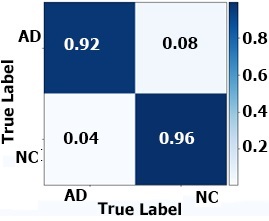 |
| ViT-TST | ViT-Bi-LSTM | CNN-TST | CNN- Bi-LSTM |
| **Figure S3.** Confusion matrices for binary classification on ADNI1: Complete 1Yr 1.5T. | | | |

### Multiclass classification

The table presents the performance of four different architectures for multiclass classification of brain images in sagittal planes, using various evaluation metrics. The CNN-Bi-LSTM, CNN-TST, ViT-Bi-LSTM, and ViT-TST architectures achieved accuracy scores of 90.24%, 87.211%, 88.824%, and 89.237%, respectively. In terms of precision, the CNN-Bi-LSTM architecture achieved the highest score of 0.91, followed by both ViT architectures with a precision score of 0.89, and finally, the CNN-TST architecture with a precision score of 0.87. In recall, the CNN-Bi-LSTM architecture achieved the highest score of 0.90, followed by the ViT-TST architecture with a recall score of 0.89, the ViT-Bi-LSTM architecture with a recall score of 0.88, and finally, the CNN-TST architecture with an F-score of 0.86. The standard deviations for all four architectures range from 1.336 to 2.157, indicating relatively small differences in performance.

Comparing the results of the four architectures, the ViT-TST architecture achieved the highest accuracy, precision, and recall scores, with an accuracy of 91.416%, precision of 0.92, and recall of 0.91. This suggests that the ViT-TST architecture is the most effective at classifying the three classes (NC, MCI, and AD) on the Complete 1Yr 1.5T. In addition, the CNN-Bi-LSTM and CNN-TST architectures achieved similar scores in all metrics, with the CNN-Bi-LSTM architecture having a slightly higher accuracy of 90.261% (±2.220) compared to the CNN-Transformer architecture with 90.174% (±2.084). This suggests that both architectures are similarly effective at classifying the three classes on the Complete 1Yr 1.5Tt. Conversely, the ViT-Bi-LSTM architecture achieved the lowest scores in all metrics, with an accuracy of 89.847% (±2.117), precision of 0.90, recall of 0.89, and F-score of 0.89. This suggests that the ViT-Bi-LSTM architecture is the least effective at classifying the three classes on the Complete 1Yr 1.5T.

Looking at the performance of the four architectures on the Complete 1Yr 1.5T dataset for multiclass classification (NC, MCI, and AD), the ViT-TST and CNN-TST architectures achieved the highest accuracy scores, both performing better than the other two architectures. The ViT-Bi-LSTM architecture also performed well with a slightly lower accuracy score than the previously mentioned architectures. The CNN-Bi-LSTM architecture had the lowest accuracy score of the four, although it still achieved a respectable score of 88.976%. The precision, F-score, and recall scores were consistent with the accuracy scores, with the ViT-TST and CNN-TST architectures achieving the highest scores and the CNN-Bi-LSTM architecture achieving the lowest scores. The results are reliable and consistent, with relatively small standard deviations for all architectures. Table S4 shows the results of multiclass classification (NC, MCI, and AD) in the sagittal, coronal, and axial planes, respectively.

**Table S5.** Results on different planes for multiclass classification ADNI1: Complete 1Yr 1.5T

| Plane | Architecture | ACC | Precision | F-score | Recall |
| --- | --- | --- | --- | --- | --- |
| Sagittal | CNN-Bi-LSTM | **90.240% (±1.769)** | 0.91 | 0.89 | 0.90 |
|  | CNN-Transformer | 87.211% (±2.157) | 0.87 | 0.86 | 0.86 |
|  | ViT-Bi-LSTM | 88.824% (±1.336) | 0.89 | 0.88 | 0.88 |
|  | ViT-Transformer | 89.237% (±1.683) | 0.89 | 0.88 | 0.89 |
| Coronal | CNN-Bi-LSTM | 90.261% (±2.220) | 0.90 | 0.89 | 0.90 |
|  | CNN-Transformer | 90.174% (±2.084) | 0.90 | 0.89 | 0.90 |
|  | ViT-Bi-LSTM | 89.847% (±2.117) | 0.90 | 0.89 | 0.89 |
|  | ViT-Transformer | **91.416% (±1.691)** | 0.92 | 0.90 | 0.91 |
| Axial | CNN-Bi-LSTM | 88.976% (±2.335) | 0.89 | 0.88 | 0.89 |
|  | CNN-Transformer | **90.218% (±1.545)** | 0.90 | 0.89 | 0.89 |
|  | ViT-Bi-LSTM | 90.131% (±2.428) | 0.90 | 0.89 | 0.90 |
|  | ViT-Transformer | 90.174% (±3.519) | 0.91 | 0.89 | 0.90 |

We also calculated each model’s classification error rate (confusion matrix). Figure S4 illustrates the confusion matrices of all the planes for multiclass classification.

**Figure S4.** Confusion matrices of sagittal planes for multiclass classification on ADNI1: Complete 1Yr 1.5T.

| Sagittal | | | |
| --- | --- | --- | --- |
| 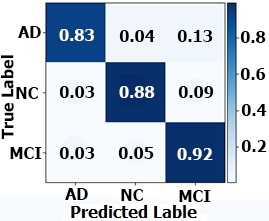 | 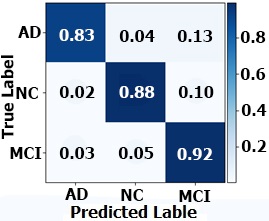 | 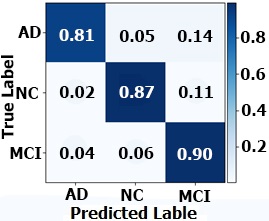 | 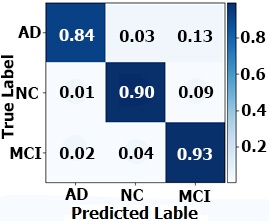 |
| Coronal | | | |
| 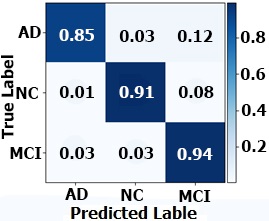 | 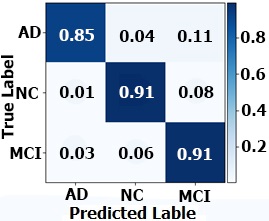 | 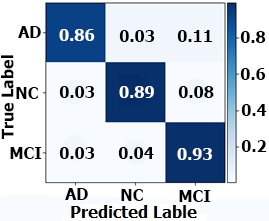 | 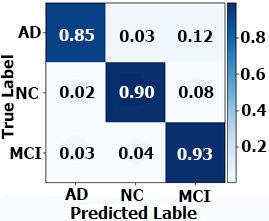 |
| Axial | | | |
| 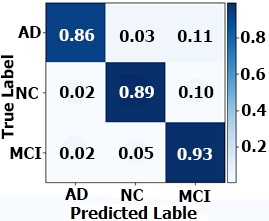 | 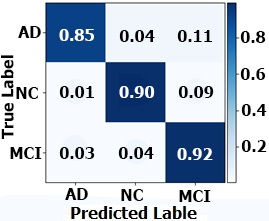 | 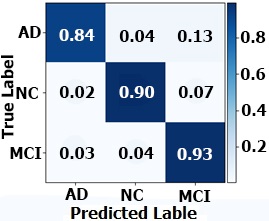 | 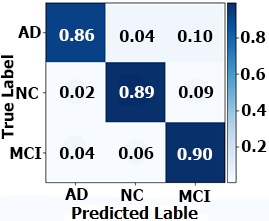 |
|  |  |  |  |
| ViT-TST | ViT-Bi-LSTM | CNN-TST | CNN-Bi-LSTM |

# References

[1] A. Vaswani *et al.*, “Attention Is All You Need,” *Adv Neural Inf Process Syst*, vol. 2017-December, pp. 5999–6009, Jun. 2017, doi: 10.48550/arxiv.1706.03762.

[2] Z. Hu, Z. Wang, Y. Jin, and W. Hou, “VGG-TSwinformer: Transformer-based deep learning model for early Alzheimer’s disease prediction,” *Comput Methods Programs Biomed*, vol. 229, p. 107291, Feb. 2023, doi: 10.1016/j.cmpb.2022.107291.

[3] Q. Wen *et al.*, “Transformers in Time Series: A Survey,” Feb. 2022, Accessed: Apr. 10, 2023. [Online]. Available: https://arxiv.org/abs/2202.07125v4
